# Neutrophil depletion reduces interstitial cajal-like cell injury and alleviates inflammation-induced motor dysfunction in guinea-pig gallbladder during acute cholecystitis

**DOI:** 10.22038/IJBMS.2022.59415.13195

**Published:** 2022-04

**Authors:** Li Zhang, Bin Yang, Yong Xiao, Bingqiang Zhang, Baoping Yu, Youlin Kuang

**Affiliations:** 1 Department of Gastroenterology, The First Affiliated Hospital of Chongqing Medical University, No. 1, Youyi Road, Yuzhong District, Chongqing 400016, China; 2 Department of Gastroenterology, Renmin Hospital of Wuhan University, No. 238, Jiefang Road, Wuhan 430060, Hubei Province, China; 3 Department of Urology, The First Affiliated Hospital of Chongqing Medical University, Chongqing, No. 1, Youyi Road, Yuzhong District, Chongqing 400016, China

**Keywords:** Common bile duct ligation, Gallbladder dysmotility, Guinea pigs, Interstitial cajal-like cells, Neutrophils, Ultrastructure features

## Abstract

**Objective(s):**

Gallbladder interstitial Cajal-like cells (ICLCs) are known as some of the players in the complex motility mechanisms affecting gallbladder motility. This study aims to explore the mechanism of guinea-pig gallbladder motility disorders during Acute Cholecystitis (AC), focusing on the relationships between neutrophil alterations, gallbladder ICLCs, and smooth muscle contractility.

**Materials and Methods:**

Forty-eight guinea pigs were randomly divided into four groups: normal, sham, common bile duct ligation (CBDL), and anti-PMN (anti-polymorphonuclear antibody treated +CBDL). Hematoxylin and eosin-stained slides from each gallbladder sample were examined for inflammation, and myeloperoxidase (MPO) activity was evaluated. The contractile response of gallbladder muscle to Ach, CCK-8, and KCl was registered by a tension transducer, and ultrastructure features of ICLCs were observed.

**Results:**

Pretreatment with anti-PMN significantly reduced the circulating neutrophils by 80% and also considerably decreased the gallbladder MPO activity by 52.9% compared with the CBDL group (*P*<0.05). After adding Ach, CCK-8, and KCl, the contraction ability in CBDL and anti-PMN groups was lower than those of normal and sham groups (*P*<0.05), and they were increased substantially in the anti-PMN group compared with the CBDL group (*P*<0.05). Transmission electron microscopy confirmed that the cytoplasm of the neutrophils was full of granules, and neutrophils contacted closely with ICLCs. The ultrastructure of ICLCs in the anti-PMN group was less inflamed and the endoplasmic reticulum was mildly dilated, and cell processes also increased.

**Conclusion:**

Anti-PMN could relieve the ultrastructure injury of ICLCs and alleviate gallbladder dysmotility during AC. Neutrophils may damage gallbladder ICLCs at first followed by dysmotility.

## Introduction

Acute acalculous cholecystitis (AC) is a pathophysiological condition characterized by gallbladder inflammation in the absence of gallstones. It is a serious illness with acute onset and progresses quickly with a mortality rate of at least 30% (1). Although its pathogenesis has not been completely elucidated, bile stasis and delayed gallbladder emptying are risk factors of AC, probably as a result of the deleterious neural and muscular actions of inflammatory mediators such as reactive oxygen species (ROS) released by polymorphonuclear neutrophils (PMN) that infiltrate heavily the gallbladder wall (2). It has been speculated that impaired muscle contractility is secondary to inflammation and it plays an important role in the clinicopathology of AC (3). Previous reports have investigated that inflammation can attenuate Ca^2+^ signals and alter the amount of contractile proteins in gallbladder smooth muscle cells (4). 

Interstitial cells of Cajal (ICC) are considered to be crucial in mediating gastrointestinal motility, they are electrically coupled to each other and neurons and myocytes. Their main roles are to initiate pacemaker activity and their dysfunction has been linked to a variety of intestinal motility disorders. The interstitial cells of Cajal-like cells (ICLCs) are also present in the gallbladder and extrahepatic biliary duct of both guinea pigs and humans (5, 6). Recent studies have shown that ICC and ICLCs frequently establish close contacts (synapses) with several types of immunoreactive cells such as lymphocytes, plasma cells, eosinophils, basophils, macrophages, mast cells, and neutrophils (7, 8). Bettolli *et al*. (9) discovered that severe inflammation results in significant ultrastructural damage of ICC networks in appendicitis, when acute inflammation subsides, ICC can recover to their normal ultrastructure. Several studies have demonstrated that damage to ICLCs in the gallbladder wall may contribute to destruction of the gallbladder motility and gallstone formation, thereby leading to the development of cholelithiasis (10, 11). Previously we have demonstrated that AC can decrease ICLCs (12) and the fact that neutrophils play an essential part in governing the loss of gallbladder ICLCs (13, 14), but the precise mechanism of gallbladder motility disorders has not been well-defined.

Our team and other colleagues have agreed that common bile duct ligation (CBDL) in guinea pigs is the most appropriate model of AC (3, 15); especially the occurrence of inflammation after CBDL is infiltrated by many inflammatory cells, primarily neutrophils, and the consequent production of inflammatory mediators, which may influence ICLCs, and contribute to the progression of motility disorder of gallbladders. It has been reported that anti-polymorphonuclear antibody (anti-PMN) treatment can efficiently reduce the number of circulating neutrophils, providing a reproducible systemic neutropenia model for the study of neutrophil function, and simultaneously result in decreased tissue neutrophils (16). The purpose of the present study was to analyze the effect of gallbladder inflammation on the integrity of ICLCs and assess the speciﬁc contribution of neutrophils by depleting them from the blood before inducing AC in guinea pigs.

## Materials and Methods

***Animals and experimental trials***

*Common bile duct ligation with and without neutrophil depletion*

Adult male guinea pigs (250–350g) were obtained from Wuhan Institute of Biological Products Company (Wuhan, China) and housed under laboratory conditions. All protocols were approved by the Institutional Animal Care and Use Committee of Wuhan University. Acute cholecystitis (AC) was induced to animals by common bile duct ligation (CBDL) for two days, as described previously (3). The animals were briefly anesthetized with an intraperitoneal injection of pentobarbital sodium (40 mg/kg body weight, Sigma Aldrich, St. Louis, MO, USA). A laparotomy was performed, and the distal end of the common bile duct was ligated (4–0 silk) with minimal manipulation of the bile duct and no manipulation of the gallbladder. The sham operation included all the surgical steps except for the common bile duct ligation. When the animals were alert, they were housed separately and provided with food and water *ad libitum*. The normal control guinea pigs, CBDL, and sham surgical control guinea pigs were all monitored until they were sacrificed two days later. 

For neutrophil depletion, animals received intraperitoneal injection of 0.8 ml rabbit anti-mouse polyclonal neutrophil serum (anti-PMN, Cedarlane Labs, Ontario, Canada) 24 hr before AC induction. Animals were randomly assigned to 4 groups: normal, sham, CBDL, and anti-PMN (anti-PMN treated +CBDL). Each group had 12 guinea pigs. Circulating PMN counts of each animal were quantified by total cell counts with a hemocytometer, followed by differential counts of Wright’s stain prepared with cytospin to determine the percentage of neutrophils. To establish the baseline circulating blood PMN counts, blood was drawn from the ear vein of each animal immediately before anti-PMN serum injection. For the anti-PMN group, at hour 24 after anti-PMN serum injection and before CBDL, another PMN count was made to assess changes in circulating blood values. Circulating blood cell counts of all groups were measured at baseline and two days later after the operation.

***Tissue preparation***

At the second laparotomy, the gallbladder was identified, removed by clamping and cutting the cystic duct. A sample of bile was obtained in order to keep a record of its color and bile volume. Blood samples were drawn for glutamic pyruvic transaminase (GPT), glutamic oxaloacetic transaminase (GOT), and bilirubin measurements. Some gallbladder samples were placed in Krebs-bicarbonate solution (KBS; see below) and processed immediately for muscle contractility studies, the others were either stored at -70 °C, examined by transmission electron microscopy, or ﬁxed in 10 % buffered formalin and then embedded in paraffin for subsequent measurement.

***Measurement of serum GOT, GPT, and bilirubin***

Whole blood samples were obtained by cardiac puncture, then centrifuged at 3,000 rpm for 5 min. The sera were collected and stored at -70 °C until assayed. Serum GOT, GPT, and bilirubin were assessed by the full automatic biochemical analysis system.

***Histopathologic study***

Freshly prepared gallbladder samples were fixed with 10% neutral formaldehyde, sectioned at 4 μm, and stained with hematoxylin and eosin. The sections underwent histopathologic analysis by light microscopy. An inflammation scoring system was used with a range from 0 (not present) to 17 (the most severe), on the basis of histologic changes as described by Parkman *et al*. (3). The degree of inflammatory cell infiltration was classified as 0, 1, 2, or 3; hemorrhage (extravasation of RBC), edema, surface ulceration, fibroblast proliferation graded as 0, 1, 2, or 3; vascular dilation and Rokitansky-Aschoff sinus formation, one of which was given a score of 1 if present and 0 if absent (3).

***In vitro muscle contractility studies***

The muscle samples were collected and placed in organ baths (7 mls) filled with KBS solution of the following composition (in mM): NaCl 120, KCl 4.6, CaCl_2_ 2.5, NaHCO_3_ 22, MgCl_2_ 1.2, NaH_2_PO_4_ 1.2, and glucose 11.5. The solution bubbled continuously with 95% O_2_ and 5% CO_2_ and the temperature was maintained at 37 °C. One side of the tissue was pinned to a hook at the bottom of the chamber and the opposite side was connected to an isometric force transducer (JZJOIH, Chengdu, China). Each muscle sample was subjected to an initial tension of 1.0 g and allowed to equilibrate for 60 min before the onset of experimental procedures. The direct effects of acetylcholine (Ach, 10^-5^mol/l), cholecystokinin octapeptide (CCK-8, 10^-6^mol/l), and potassium chloride (KCl, 60mmol/l) on gallbladder tone were examined. The mean contraction level was recorded as control value and the effects of drugs (Ach, CCK-8, or high K^+^) as the response value. Analyses were based on the maximal values of contractions and the results were presented as the change rate (R), where R = [(response maximal value - control value)/control value].

***Measurement of myeloperoxidase (MPO) activity***

Neutrophil migration to the gallbladder was evaluated using an MPO kinetic-colorimetric assay, as described by the manufacturer (Nanjing Jiancheng Bioengineering Institute, China). Tissue samples were weighed, fixed with 19-fold phosphate buffer two, and homogenized. Afterward, 0.9 ml homogenate added 0.1 ml buffer three, homogenized, and incubated at 37 °C for 15 min. According to the protocol, buffer four, TMB Substrate, and buffer six were fixed in the ration, mixed, and left in a 60 °C water bath for 10 min. The levers of MPO of gallbladder tissues were detected in 460 nm absorbance values.

***Transmission electron microscopy***

Tissues were placed in 2.5% glutaraldehyde in 0.1 mol/l phosphate buffer (PB) pH 7.4 overnight at 4 °C. They were then washed in 0.1 mol/l PB (2×15 min) and post-fixed with 2% osmium tetroxide for 2 hr at 4 °C. After another wash with 0.1 mol/l PB, they were dehydrated in a series of graded ethanol (50, 75, 95, and 2 × 100%, 15–20 min each) and immersed in a mixture (1:1) of propylene oxide and Epone 812 resin overnight, and then embedded in Epon 812. Ultra-thin sections were produced using a Reichert OMU4 ultramicrotome with a diamond-cutting blade. Sections were mounted on copper grids, stained with 2% uranyl acetate in 30% ethanol and lead citrate, and then kept under observation under an OPTON EM10C transmission electron microscope.

***Statistical analysis***

All data were presented as mean ± SEM. One-way analysis of variance or Student’s t-test was used to determine the difference between the means of different groups. *P*<0.05 was considered to be a statistically signiﬁcant difference.

## Results

***Laboratory measurements***

**Figure 1:**
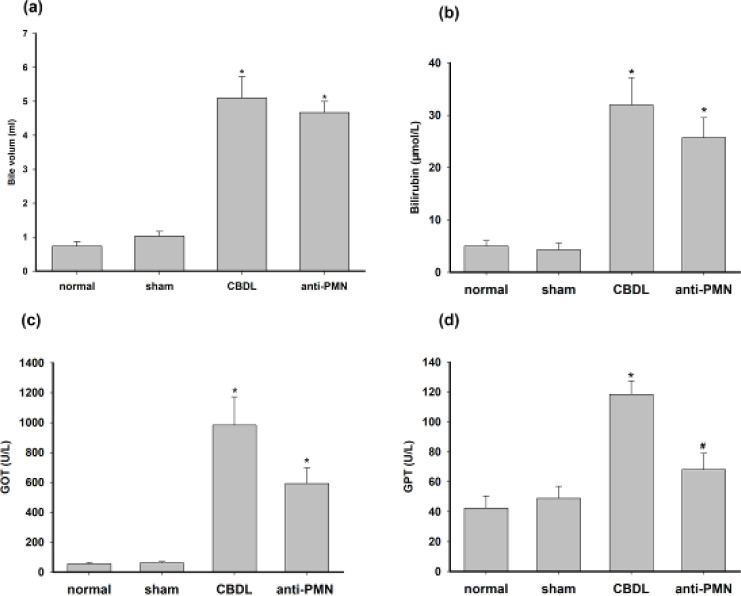
Gallbladder volume, laboratory measurements of our guinea pig model of AC. (a) Bile volume in the gallbladder from guinea pigs. (b) Level of bilirubin in serum from guinea pigs. (c) Level of GOT in serum from guinea pigs. (d) Level of GPT in serum from guinea pigs. Data shown are the mean ± SD (n=12/group). * *P*<0.05 vs sham, normal; # *P*<0.05 vs CBDL

The color of the bile was the golden-brown color before CBDL (including normal and sham groups) and changed to dark-green after CBDL (including CBDL and anti-PMN groups). Compared with normal and sham groups, the bile volume and the level of GOT and bilirubin in serum significantly increased (*P*<0.05) in CBDL and anti-PMN groups, but there was no statistically significant difference between CBDL and anti-PMN groups (*P*>0.05). However, the Anti-PMN treatment decreased the level of GPT compared with the CBDL group (Figure 1).

***Anti-PMN treatment reduces circulating neutrophils***

**Figure 2:**
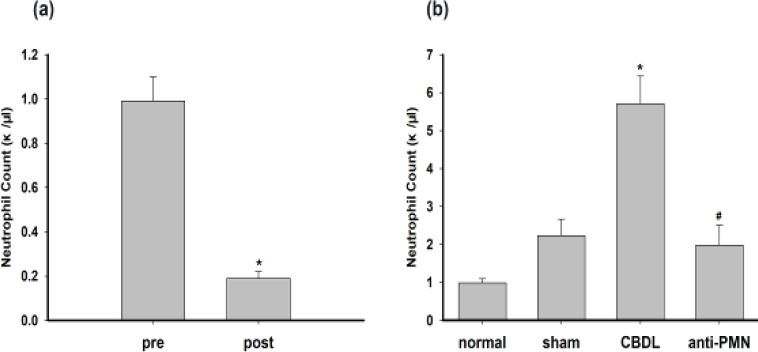
Neutrophils in peripheral blood from guinea pigs. (a) pre (before anti-PMN serum injection) and post (24 hr after anti-PMN serum injection) neutrophil count from guinea pigs, *P<0.05 vs pre; κ/μl=10^3^/μl. (b)neutrophil count in all group;*P<0.05 vs normal, sham; #*P*<0.05 vs CBDL, κ/μl=10^3^/μl

Blood was drawn from all 48 animals to determine the baseline circulating cell counts. The mean neutrophil count in the anti-PMN-treated animals decreased from 0.99 ±0.11 ×10^3^ cells/μl at baseline to 0.19±0.03 ×10^3^ cells/μl just before CBDL, representing an 80% decrease in the absolute neutrophil number (*P*<0.05). After the CBDL, the absolute neutrophil count in the CBDL group was remarkably higher than those in normal, sham, and anti-PMN groups (*P*<0.05). Compared with the CBDL group, the number of circulating neutrophils decreased notably in the anti-PMN group. (5.71±0.73 ×10^3^ cells/μl vs 1.98±0.53 ×10^3^ cells/μl, n=12/group, *P*<0.05) (Figure 2).

***Anti-PMN treatment reduces infiltrating neutrophils and inflammation***

**Figure 3:**
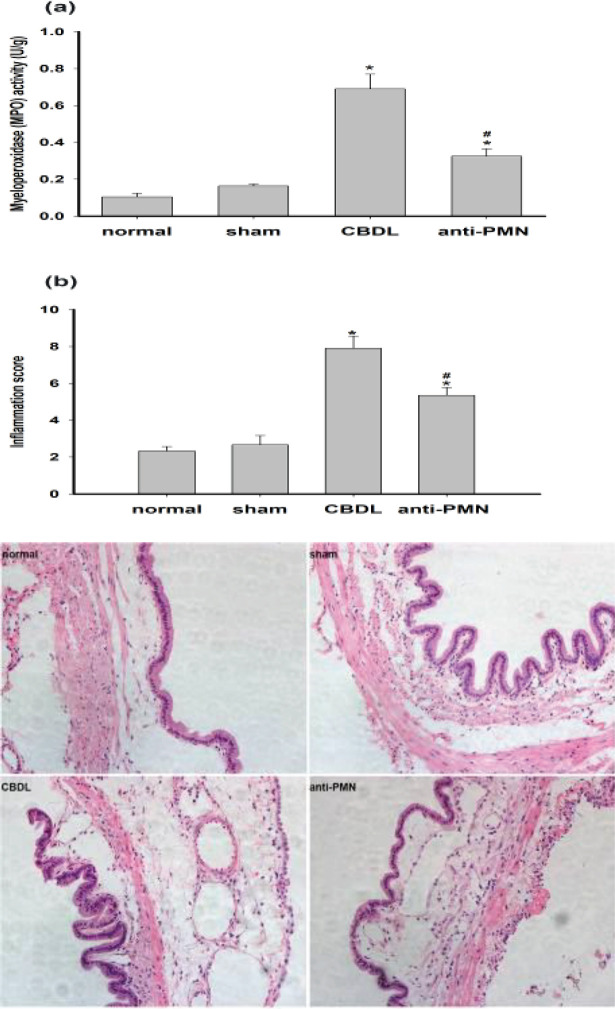
MPO, gallbladder inflammation scores, and histopathological analysis in each group. (a) MPO activity in the gallbladder from guinea pigs. (b) Inflammation scores and photomicrographs of gallbladder samples stained with hematoxylin and eosin in each group. Magnification of photomicrographs is ×200. Data shown are the mean ± SD (n=12/group). *P<0.05 vs normal, sham; #*P*<0.05 vs CBDL

The level of neutrophil infiltration was quantified using MPO as a marker. When compared with the normal or sham groups, bile duct ligation resulted in significant increases in the MPO activity in the gallbladder (normal: 0.1039±0.02 U/g vs CBDL: 0.6907±0.08 U/g, normal: 0.1039±0.02 U/g vs anti-PMN: 0.3252±0.04 U/g and sham: 0.1637±0.01 U/g vs CBDL: 0.6907±0.08 U/g, sham: 0.1637±0.01 U/g vs anti-PMN: 0.3252±0.04 U/g, n=12/group, *P*<0.05). Pretreatment of animals with anti-PMN significantly reduced the gallbladder MPO activity by 52.9% in comparison with the CBDL group (Figure 3).

We and others have shown an early and prominent inflammation in this CBDL model (3, 4). As shown in Figure 3, inflammatory cells infiltrated the wall of the gallbladder of the CBDL group, with some splaying of the muscle fibers due to edema, as well as vascular dilation and fibroblast proliferation. The mean inflammation score in the CBDL group was higher than those of the normal and sham groups (normal: 2.33±0.25 vs CBDL: 7.9±0.67 and sham: 2.67±0.5 vs CBDL 7.9±0.67, n=12/group, *P*<0.5). In the anti-PMN group, the score was significantly lower compared with CBDL groups (anti-PMN: 5.35±0.43 vs CBDL: 7.9±0.67, n=12/group,* P*<0.05) (Figure 3).

There was no significant inflammatory cell infiltrate in the normal and sham groups. By contrast, the thickness of the gallbladder wall from the CBDL animals was increased due to the presence of edema, vascular dilation, and white blood cell infiltration (primarily neutrophils). The mean score for inflammation in the CBDL group was higher than those in the corresponding normal (2.33±0.25) and sham groups (2.67±0.5) (*P*<0.05). In the anti-PMN group, the score was significantly decreased compared with the animals from the CBDL group (*P*<0.05). There was no statistically significant difference between the inflammation score of the normal groups when compared with the sham groups.

***Neutrophil depletion reduces ICLCs injury***

Transmission electron microscopy (TEM) confirmed the presence of ICLCs in the muscular layers in the gallbladder. In the normal and sham groups, the cell morphology was characterized by an ovoid cell body with multiple, thin processes and large prominent nuclei with sparse perinuclear cytoplasm. Numerous caveolae and mitochondria are present, and ICLCs were in direct contact with other ICLCs or gallbladder smooth muscle cells (GBSM). However, ICLCs in the CBDL group sustained major structural changes – they became swollen with a dilated endoplasmic reticulum, and the electron density of the perinuclear cytoplasm was significantly reduced. The caveolae were lined in the membrane and many processes were ruptured, with complete loss of their cytoplasmic contents. The contact between the two ICLCs and between ICLCs and GBSM cells was disrupted. Furthermore, extensive synaptic connections between ICLCs and the neutrophils in the gallbladder were also observed. Neutrophils were filled with a large number of granules in the cytoplasm, which were closely connected with the damaged ICLCs body and processes, and even engulfed ICLCs.

**Figure 4:**
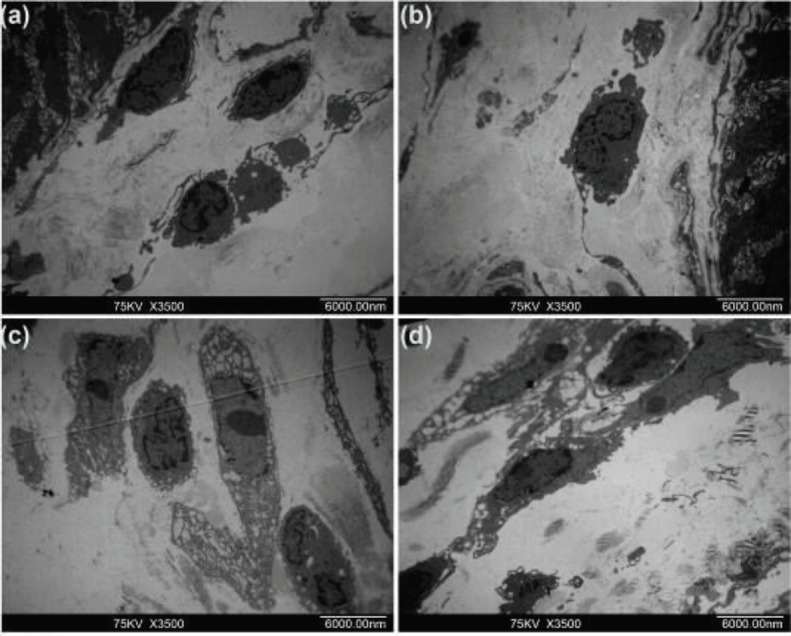
ICLCs ultrastructural features. (a), (b) show ICLCs of the normal and sham group, forming intact network-like structures by connecting with other ICLCs through long cellular processes. (c) shows ICLCs of CBDL group, formation of the network-like structures of the ICLCs was impaired, as highlighted by the shortening of the ICLCs. (d) shows ICLCs of the anti-PMN group, compared with that in the CBDL group, a few of the network-like structures were observed

**Figure 5:**
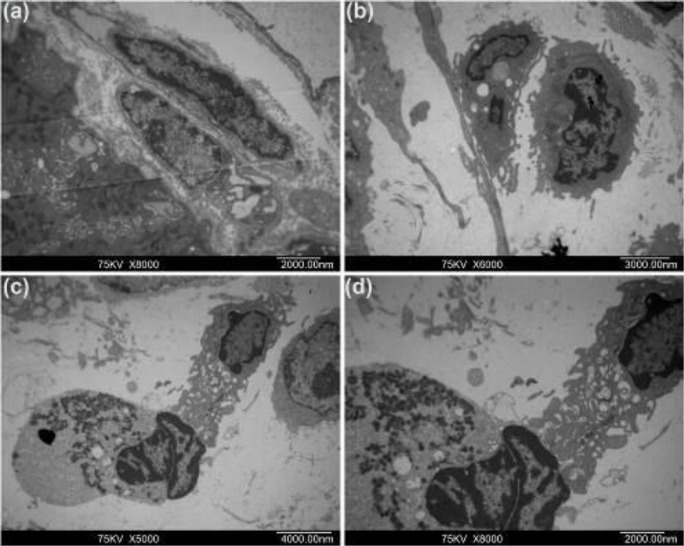
ICLCs ultrastructural features. (a) In the sham group, gallbladder ICLCs showed large prominent nuclei with sparse perinuclear cytoplasm. Numerous caveolae and mitochondria are present. (b) In the CBDL group, ICLCs were swollen, endoplasmic reticulum dilatation, and signiﬁcant reduction in electron density of the perinuclear cytoplasm. (c)and (d) show that in the CBDL group neutrophils were filled with a large number of granules in the cytoplasm, which were closely connected with the damaged ICLCs body and processes, and then engulfed ICLCs

After anti-PMN treatment, the structural changes of ICLCs persisted in both perinuclear cytoplasm and the processes. But compared with the CBDL group, there was less swelling, and the dilation of the endoplasmic reticulum was mild. Membrane-to-membrane contacts were frequently seen to be preserved or even to be strengthened slightly between two ICLCs and between ICLCs and GBSM cells (Figures 4, 5).

***Neutrophil depletion alleviates the contractibility of gallbladder dysfunction***

**Figure 6:**
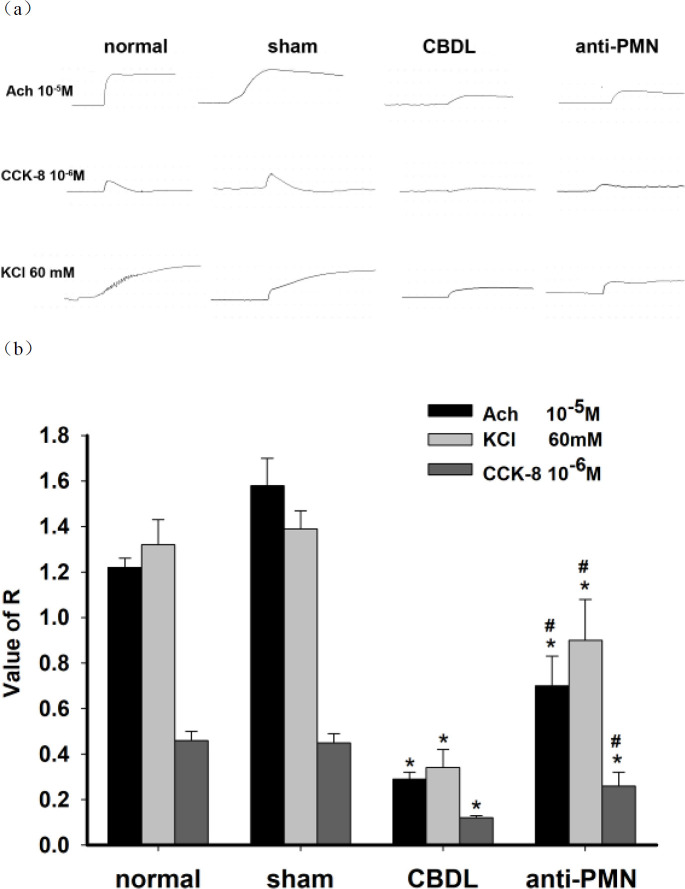
Contractility of gallbladder muscle strips in each group. (a) Representative recording of the effect of Ach (10-5M), CCK-8(10-6M), and KCl (60 mM) on the contraction of the gallbladder muscle strips in each group. (b) Summarized results of the contractile responses induced by Ach (10-5M), CCK-8(10-6M), and KCl (60mM). Data shown are the mean ± SD (n=12/group). *P<0.05 vs normal, sham; #*P*<0.05 vs CBDL

The contractile activities of gallbladder muscle in the CBDL group and the anti-PMN group were signiﬁcantly decreased when compared with that of the normal or sham group. After adding Ach, CCK-8 or KCl, the R values of the muscle strips in CBDL and anti-PMN groups were notably less than those in normal and sham groups (Ach: 0.29±0.03, 0.70±0.13, 1.22±0.04, 1.58±0.12, respectively; CCK-8: 0.12±0.01, 0.26±0.06, 0.46±0.04, 0.45±0.04, respectively; KCl: 0.34±0.08, 0.90±0.18, 1.32±0.11, 1.39±0.08, respectively; Figure 5). However, the contractile response to Ach, CCK-8, and KCl was obviously elevated in the anti-PMN group compared with the CBDL group, respectively (Ach: 0.70±0.13 vs 0.29±0.03, CCK-8: 0.26±0.06 vs 0.12±0.01, KCl: 0.90±0.18 vs 0.34±0.08, n=12/group, *P*<0.05). Thus, this dysfunction of gallbladder contractibility can be partially reversed by neutrophil depletion (Figure 6).

## Discussion

This paper has indicated that anti-PMN treatment can greatly inhibit peripheral blood neutrophils and reduce those infiltrating the gallbladder after CBDL, with several salient outcomes: (1) Anti-PMN treatment could reduce both circulating and infiltrating neutrophils and attenuate gallbladder inflammation. (2) The morphology of ICLCs was altered by CBDL but displayed ultrastructural recovery after neutrophil depletion. (3) Neutrophil depletion alleviated inflammation-induced motor dysfunction. Together, these results suggest that neutrophils are involved in ICLCs injury and gallbladder dysfunction in experimental AC.

Studies have shown that leukocytosis and abnormal liver tests (aminotransferases, alkaline phosphatase, and bilirubin) are not speciﬁc to AC patients, while older patients with a high WBC count are more prone to severe gallbladder complications, such as gangrene, perforation, and abscesses (1, 17). Parkman *et al*. indicated that CBDL produced progressive gallbladder distension, both visually and as indicated by the increase in gallbladder volume (3, 18). Soylu *et al*. reported that blood levels of bilirubin, glutamic pyruvic transaminase (GPT), and alkaline phosphatase (ALP) were all escalated after bile duct ligation in all CBDL animals (18). The results of the present study demonstrated that the bile volume and the level of GOT, and bilirubin in serum significantly increased, which was consistent with these reports. Both GPT and GOT are suitable indicators for evaluating liver dysfunction. Under normal circumstances, GPT activity was primarily concentrated in the cytosol, while GOT activity was mostly located in mitochondria. During hepatic damage, the level of the mitochondrial isoenzyme GOT increases to a greater extent in acute liver diseases to reflect a higher degree of liver dysfunction (19). In the present study, although anti-PMN treatment decreased the level of GPT compared with the CBDL group, GOT level remained elevated in the sera. It meant that the liver was still severely damaged. The main mechanisms are probably increased permeability of the hepatocyte membrane caused by elevated pressure in the bile ducts, combined with a direct toxic effect of retained bile acids (20).

 It is believed that PMN is the chief immune cell population in the circulating blood and constitutes an important component in the first line of defense following infection or tissue injury (21). Activated PMNs were shown to produce and release reactive oxygen species (ROS), inflammatory cytokines, directly inducing tissue damage. Therefore, PMN accumulation in tissues often suggests acute inflammatory responses (21). MPO is the main component of the azurophilic granules of the PMN and is the standard marker used when detecting infiltrated neutrophils. It has been discovered that minocycline, a type of broad-spectrum anti-inflammatory antibiotic reduced neutrophil infiltration after intracerebral hemorrhage, as it acts on several cell types with intricate actions. These outcomes cannot simply be ascribed to decreased neutrophil entry. Therefore, researchers adopted the use of anti-neutrophil serum (anti-PMN) to induce neutropenia and assessed the roles of PMN in response to brain injury more closely (16). Similar approaches are taken in the present paper to deplete PMN from the blood. The guinea pigs became clinically neutropenic, and the 80% reduction in blood neutrophils was comparable to previous studies in other animal models (16, 22). It was discovered that CBDL significantly increased the infiltration of neutrophils in the gallbladder wall. However, the gallbladder MPO activity after anti-PMN treatment was reduced by 52.9%, and the histopathologic examination also revealed a lower inflammation score. These findings provide evidence that administration of anti-PMN antibody significantly inhibited neutrophil function and attenuated gallbladder inflammation during AC.

It is now generally accepted that ICCs generate pace-making slow waves and regulate the rhythmic smooth muscle contraction in the gastrointestinal tract. Studies on ICCs and inflammation carried out using animal models with acute bacterial infections and in human inflammatory bowel disease (IBD) demonstrated that inflammation affects ICCs. These reports also indicated that intestinal inflammation impairs the function and structure of ICCs (9). ICLCs are present in the gallbladder wall and subsequent studies have suggested that ICLCs could be participating in the regulation of gallbladder motility (10, 12, 23). Intracellular Ca^2+^ in gallbladder ICLCs is a key factor in gallbladder contraction. We previously showed that calcium transient in gallbladder ICLCs is impaired when ICLCs are cocultured with neutrophils (24). During inflammatory conditions, immune cells produce inflammatory cytokines, such as interferon-gamma (IFN-γ), interleukin-1 beta (IL-1β), tumor necrosis factor-alpha (TNF-α), and IL-6. In cell cluster systems, treatment with individual pro-inflammatory cytokines (TNF-α, IL-1β, and IL-6) does not impair the pacemaker activity of ICC. While oxidative stress decreases the expression of ICC markers and impairs the pacemaker function of ICCs (25). We and other researchers have shown that inflammatory cells infiltrating the gallbladder are primarily neutrophils (3, 12, 13, 15, 24). It has also been established that ROS released by infiltrating PMN or generated within the muscle cells are significantly increased in gallbladder specimens affected by AC, and ROS contributes to gallbladder injury (2). ICC may be susceptible to ROS-induced oxidative stress under inflammatory conditions (25). In our study, distribution of ICLCs in the gallbladder muscle similar to other recent reports was described (3, 12, 13), but ICLCs in AC were inflamed and had low cytoplasmic contrast. Furthermore, extensive synaptic connections between the neutrophils and ICLCs in the gallbladder were also observed after CBDL, which indicated that under disease conditions neutrophils accumulate around the ICLCs, which are sensitive to injury in this inflammatory state. The neutrophils could secrete ROS, then cause damage to the structure and function of the gallbladder ICLCs. It is also observed that neutrophil-mediated ICLCs injury occurs possibly via phagocytosis, while other scholars argue that intestinal ICCs have a phagocytic-like property (26). In addition to this direct impact on ICLCs, neutrophils also cause indirect damage through down-regulation of SCF, c-kit, which play a role in ICLC survival, proliferation, or function (12, 14). 

 In the current study, the ultrastructural damage of gallbladder ICLCs was remedied by neutrophil depletion. Meanwhile, it was noticed that CCK-, Ach-, and KCl-induced gallbladder contraction was decreased in CBDL induced AC, and this could also be partially reversed by neutrophils depletion. It has been reported that severe inflammation results in significant ultrastructural damage of nerves and ICC networks in appendicitis. When ICC recovered and the nerve’s ultrastructure normalized, slow-wave electrical activity and intestinal motility may recover as well (9). It is possible that neutrophil depletion reduced neutrophil infiltration and attenuated the gallbladder inflammation, then recovered ICLCs ultrastructure, and finally alleviated the inflammation-induced motor dysfunction in the gallbladder during AC. 

It is also vital to mention that gallbladder smooth muscle contraction can be directly stimulated by CCK via signaling through CCK-A receptors in the gallbladder ICLCs (10, 27). Many studies have shown that acute gallbladder inflammation with CBDL affects muscle contraction. It impairs the response to agonists that act on membrane constituents such as CCK-8 and acetylcholine (Ach) which stimulate transmembrane receptors and KCl which opens calcium channels (2, 3, 4, 28). Cholecystitis can decrease both Ca^2+^release and Ca^2+^influx in gallbladder smooth muscle and cause gallbladder motility disorders (4). There is also obstructive jaundice with accumulation of bilirubin, bile salts, and other toxins that are usually excreted into bile and may affect smooth muscle contractility. If treated with the anti-inflammatory agent, indomethacin, in the early stages of CBDL, gallbladder motor dysfunction can be improved (3). Our previous study indicated that development of gallbladder hypomotility involves neutrophils (24). In this study, inflammatory cells infiltrating the gallbladder are primarily neutrophils. anti-PMN treatment could attenuate gallbladder inflammation, and this dysfunction of gallbladder contractibility can be partially reversed. We speculate that in our experimental model of AC with CBDL, the inflammatory changes, primarily neutrophils, are responsible for the contractile abnormalities.

Neutrophils can produce inflammatory mediators that help recruit monocytes/macrophages, thus amplifying the inflammatory response (16). After injury, when excess immunocytes are present in the tissue, these inflammatory cells could negatively affect the ICLCs, impair their pacemaker function and the contractibility of the gallbladder, resulting in pathogenesis. Anti-PMN could decrease the neutrophil infiltration in gallbladder tissues and reduce inflammation in the AC animal model. Anti-PMN could also alleviate the ultrastructure injury of ICC and elevate the contractile response to Ach, CCK-8, and KCl on gallbladder muscle strips. Neutrophils may damage gallbladder ICC firstly, then show impaired contraction of gallbladder smooth muscle.

## Conclusion

It has been demonstrated in this paper that neutrophils can greatly affect ICLC ultrastructure in a guinea pig model of AC. Neutrophil depletion reduces ICLCs injury and alleviates the inflammation-induced motor dysfunction in the gallbladder. These findings imply the potential application of neutrophils–immunocyte‐targeted drug treatments for patients suffering from AC.

## Authors’ Contributions

LZ and BY Conceived and designed the experiments. LZ, BY, YX, and YK Performed the experiments. LZ, BZ, and YK Wrote the manuscript.

## Conflicts of Interest

All authors declare that there are no conflicts of interest.
